# Effect of vitamin D and calcium levels on gender differences in bone minerals: A cross-sectional study based on NHANES 2011 to 2018

**DOI:** 10.1097/MD.0000000000046008

**Published:** 2025-11-14

**Authors:** Kai Chen, Pengli Zhang

**Affiliations:** a Department of Orthopedic Spine Surgery, Tongde Hospital of Zhejiang Province, Hangzhou, Zhejiang, China.

**Keywords:** bone health, bone mineral content, bone mineral density, calcium level, gender difference, vitamin D

## Abstract

Bone health, assessed by bone mineral content (BMC) and bone mineral density (BMD), is a critical determinant of overall health and quality of life. Vitamin D and calcium are well-established factors influencing bone metabolism, but their effects may differ by gender and remain insufficiently clarified. This study aimed to examine the associations of vitamin D levels and total calcium concentrations with BMC and BMD, with a focus on gender-specific effects. Based on health data gathered from 2011 to 2018, this cross-sectional study included 11,246 participants aged ≥18 years. The study variables included BMC, BMD, 25-hydroxyvitamin D_2_ + D_3_ [25(OH)D_2_ + D_3_], 25(OH)D_2_, 25(OH)D_3_, and total calcium levels, with adjustments for covariates such as smoking, alcohol consumption, and body mass index. The associations of vitamin D and calcium with BMC and BMD were evaluated using multivariate linear regression, followed by gender-specific subgroup analyses. The results of this study disclosed that both vitamin D levels and total calcium concentrations were significantly associated with BMC and BMD (*P* < .05). Subgroup analysis revealed that, in males, vitamin D levels – including 25(OH)D_2_+ D_3_, 25(OH)D_2_, and 25(OH)D_3_ – were positively associated with BMC and BMD, while total calcium showed no significant effect. In contrast, among females, vitamin D levels were not significantly associated with BMC or BMD, whereas total calcium levels showed significant associations with both (*P* < .05). Both vitamin D and calcium are essential for bone health maintenance, and their effects differ significantly between genders. Vitamin D levels have a stronger impact on bone health in males, while calcium intake is more influential in females. This observation may inform gender-specific nutritional interventions and offers theoretical support for personalized health approaches.

## 1. Introduction

Bone health is widely regarded as a critical indicator of overall health and quality of life, especially among older adults. Bone mineral density (BMD) and bone mineral content (BMC) are pivotal indices used to assess bone quality and fracture susceptibility.^[1,2]^ Osteoporosis is a progressive bone disease marked by low BMD and deterioration of bone microarchitecture, leading to a significantly elevated risk of fractures and disability. Notably, osteoporosis has become a prominent global health issue. According to estimates by the World Health Organization, osteoporosis affects hundreds of millions of individuals worldwide, with particularly high prevalence and disability rates among the elderly.^[3–5]^ In light of rapid population aging, developing effective strategies for the prevention and management of osteoporosis has emerged as a critical concern for researchers and public health professionals.

Among the various factors influencing bone health, vitamin D and calcium play particularly critical roles. Calcium constitutes the primary mineral component of bone, while vitamin D facilitates intestinal calcium absorption and bone mineralization, thereby contributing to the maintenance of calcium-phosphorus homeostasis.^[6–8]^ As the principal biomarker for assessing vitamin D status, 25-hydroxyvitamin D [25(OH)D] primarily comprises 2 forms: 25(OH)D_2_ and 25(OH)D_3_.^[9,10]^ Although numerous studies have demonstrated a close association between vitamin D and bone health, the effects of different forms of 25(OH)D on BMD and BMC remain controversial. Previous studies have disclosed a positive correlation between 25(OH)D_3_ levels and BMD, whereas the physiological role of 25(OH)D_2_ remains unclear. Considerable controversy still exists regarding the effects of different forms of vitamin D.^[11,12]^ In addition, vitamin D deficiency is widely recognized as a key risk factor for osteoporosis and increased fracture risk; however, sensitivity to vitamin D may vary substantially across populations, particularly by race and gender.^[13,14]^ For instance, studies have disclosed a stronger association between vitamin D levels and BMD in non-Hispanic White population compared with African Americans.^[15]^ Nevertheless, few large-scale population studies have systematically assessed the independent effects of different forms of 25(OH)D on bone health, and gender-specific effects remain largely unexplored.

Recent research has highlighted pronounced gender-specific regulatory mechanisms in bone metabolism. In males, androgen and vitamin D levels play a more prominent role, whereas in females, particularly those who are postmenopausal, bone metabolism appears more dependent on estrogen and calcium homeostasis.^[16,17]^ In addition, lifestyle factors such as smoking, alcohol consumption, and dietary habits have been shown to significantly affect bone health, potentially exerting synergistic or antagonistic interactions with nutritional factors.^[18,19]^ Therefore, a comprehensive evaluation of the gender-specific effects of vitamin D, calcium, and other demographic and lifestyle factors is essential for the development of targeted interventions to promote bone health.

The National Health and Nutrition Examination Survey (NHANES) database offers representative and widely applicable data on the U.S. population, allowing for comprehensive analysis of bone health and its associated factors at the population scale, thereby holding significant relevance for public health.^[20]^ To date, few studies have employed NHANES data to thoroughly investigate the gender-related effects of vitamin D and calcium on bone health, particularly with respect to the distinct roles of of 25(OH)D_2_, 25(OH)D_3_ and total calcium intake in males and females.

Accordingly, based on NHANES data from 2011 to 2018, this study aimed to systematically examine the gender-specific effects of 25(OH)D_2_, 25(OH)D_3_, total calcium intake, and other demographic and lifestyle factors on BMD and BMC using large-scale population data. Through a series of investigations, this study sought to provide more precise evidence to support the precision prevention of osteoporosis and the improvement of bone health in male and female populations.

## 2. Methods

### 2.1. Data sources and study design

The data used in this study were obtained from the NHANES, a nationally representative, continuous cross-sectional survey established by the Centers for Disease Control and Prevention in the United States. NHANES employed a multistage, stratified, random sampling design to collect data on the health, nutritional status, and disease conditions of the non-institutionalized population across the United States. Data for this study were extracted from the publicly released health interview and examination data of NHANES 2011 to 2018, with particular emphasis on BMC, BMD, total vitamin D [25(OH)D_2_ and 25(OH)D_3_], and total calcium.

### 2.2. Sample inclusion and exclusion criteria

A total of 39,156 participants were included in the NHANES 2011 to 2018 database. To ensure analytical rigor and accuracy, this study applied the following inclusion and exclusion criteria.

Inclusion criteria included: age ≥ 18 years; complete data on BMC and BMD; at least one available vitamin D value [25(OH)D_2_ or 25(OH)D_3_] data; and complete total calcium measurements. As for exclusion criteria, participants missing any of the aforementioned key variables were excluded.

**Figure 1.**
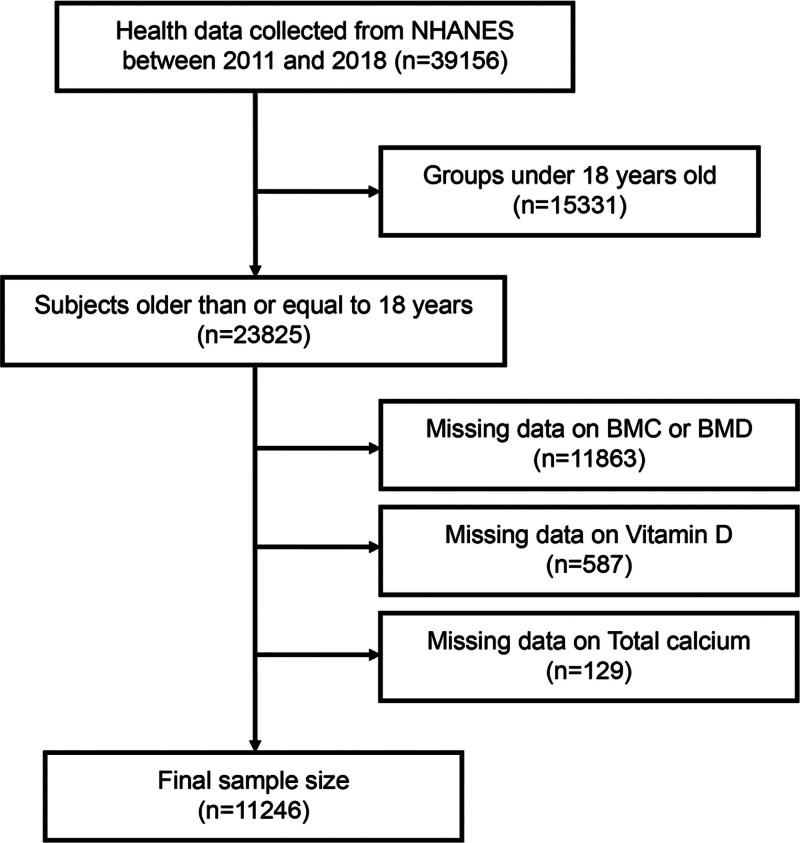
Sample selection flowchart.

Following strict screening procedures, 11,246 individuals were retained for final analysis (Fig. 1).

### 2.3. Measurement of main study variables and covariates

Key variables in this study included BMC, BMD, vitamin D levels, and total calcium levels. BMC (g) and BMD (g/cm^2^) were measured using dual-energy X-ray absorptiometry. Vitamin D [25(OH)D_2_ and 25(OH)D_3_, nmol/L] was measured via ultra-high performance liquid chromatography tandem mass spectrometry method, and total vitamin D was calculated as the sum of 25(OH)D_2_ and 25(OH)D_3_. Total serum calcium (mmol/L) was measured with the Beckman Synchron LX20 automated chemistry analyzer.

In addition, based on previous literature,^[20]^ this study involved following demographic characteristics: age, gender, race (Mexican American, non-Hispanic White, non-Hispanic Black, other Hispanics, and other races), education background, and marriage status, as well as smoking, alcohol consumption (drinking vs non-drinking), and body mass index (BMI). These bone health-related variables were incorporated into the analysis as key covariates to reduce potential confounding.

### 2.4. Statistical analysis

The baseline characteristics of the study population were analyzed through descriptive statistics. Continuous variables were expressed as mean ± standard deviation, and categorical variables were presented as frequencies and percentages. The Shapiro–Wilk test was used to assess data normality. If data were non-normally distributed, natural logarithmic transformation was applied to improve normality and homogeneity of variance. To further clarify sample distribution differences under varying bone health statuses, participants were classified into high and low content groups based on the median values of BMC and BMD, followed by intergroup statistical testing.

Multivariate linear regression models were used to evaluate the effects of vitamin D and calcium levels on BMC and BMD, while adjusting for potential confounders including age, gender, race, BMI, smoking, and alcohol consumption. Gender-specific analyses were further conducted in male and female subgroups.

To ensure the absence of severe multicollinearity among variables in the regression model, the variance inflation factor was used for collinearity diagnostics. All variables had variance inflation factor values <5, suggesting that no significant multicollinearity was present in the model. In addition, the sensitivity analysis demonstrated that removing outliers for BMI and vitamin D had no substantial impact on the overall trend or statistical significance, thereby supporting the stability of the study results. All statistical analyses were performed using R software (version 4.2.2). Two-sided tests were applied to all data analysis, and a *P*-value < .05 was considered statistically significant.

## 3. Results

### 3.1. Population baseline characteristics

A total of 11,246 participants were included in this study, with a mean age of 37.7 ± 12.4 years. Of the participants, 49.6% were male and 50.4% were female. The mean BMI for the overall population was 28.7 ± 6.93 kg/m^2^. Detailed descriptive statistics for all variables were presented in Table 1. Participants were grouped based on the median values of BMD and BMC. The proportion of males in the high BMD group was significantly higher than that of females (61.8% vs 38.2%, *P* < .001), and the difference was more pronounced in the high BMC group (74.4% vs 25.6%, *P* < .001). Moreover, significant differences were observed between groups in terms of age, race, BMI, education background, marriage status, smoking, and alcohol consumption (*P* < .05; Tables 1 and 2).

**Table 1:** Baseline characteristics of participants stratified by bone mineral density (BMD) levels.

	Total (n = 11,246)	High (n = 5651)	Low (n = 5595)	*P*-value
Age (yr)	37.7 ± 12.4	37.1 ± 11.7	38.4 ± 12.9	<.001
Gender				
Male	5576 (49.6%)	3494 (61.8%)	2082 (37.2%)	<.001
Female	5670 (50.4%)	2157 (38.2%)	3513 (62.8%)	
BMI (kg/m^2^)	28.7 ± 6.93	29.6 ± 6.90	27.8 ± 6.84	<.001
Education level				
<High school	1911 (17.0%)	895 (15.8%)	1016 (18.2%)	.004
Complete High school	2587 (23.0%)	1309 (23.2%)	1278 (22.8%)	
>High school	6746 (60.0%)	3447 (61.0%)	3299 (59.0%)	
Race				
Mexican American	1783 (15.9%)	783 (13.9%)	1000 (17.9%)	<.001
Non-Hispanic White	3864 (34.4%)	1900 (33.6%)	1964 (35.1%)	
Other Hispanic	1189 (10.6%)	490 (8.67%)	699 (12.5%)	
Non-Hispanic Black	2295 (20.4%)	1631 (28.9%)	664 (11.9%)	
Other Race	2115 (18.8%)	847 (15.0%)	1268 (22.7%)	
Marriage status				
Married/Living with partner	6226 (60.0%)	3066 (58.4%)	3160 (61.7%)	.001
Widowed/Divorced/Separated/Never married	4143 (40.0%)	2182 (41.6%)	1961 (38.3%)	
Alcohol				
No	9384 (91.0%)	4641 (90.1%)	4743 (91.8%)	.003
Yes	930 (9.02%)	508 (9.87%)	422 (8.17%)	
Smoke				
Never	6886 (62.5%)	3339 (60.3%)	3547 (64.8%)	<.001
Former	1759 (16.0%)	920 (16.6%)	839 (15.3%)	
Current	2366 (21.5%)	1275 (23.0%)	1091 (19.9%)	
25(OH)D_2_ + D_3_ (nmol/L)	60.1 ± 24.9	59.4 ± 24.3	60.9 ± 25.5	.001
25(OH)D_2_ (nmol/L)	3.14 ± 9.11	3.06 ± 9.14	3.22 ± 9.08	.352
25(OH)D_3_ (nmol/L)	57.0 ± 24.8	56.3 ± 24.1	57.7 ± 25.6	.003
Total calcium (mmol/L)	2.34 ± 0.09	2.35 ± 0.09	2.34 ± 0.09	.016

Data were presented as mean ± SD or n (%).

BMI = body mass index.

**Table 2:** Baseline characteristics of participants stratified by bone mineral content (BMC) levels.

	Total (n = 11,246)	High (n = 5623)	Low (n = 5623)	*P*-value
Age (yr)	37.7 ± 12.4	37.2 ± 12.7	38.3 ± 12.7	<.001
Gender				
Male	5576 (49.6%)	4181 (74.4%)	1395 (24.8%)	<.001
Female	5670 (50.4%)	1442 (25.6%)	4228 (75.2%)	
BMI (kg/m^2^)	28.7 ± 6.93	30.1 ± 7.09	27.3 ± 6.47	<.001
Education level				
<High school	1911 (17.0%)	876 (15.6%)	1035 (18.4%)	<.001
Complete High school	2587 (23.0%)	1330 (23.7%)	1257 (22.4%)	
>High school	6746 (60.0%)	3417 (60.8%)	3329 (59.2%)	
Race				
Mexican American	1783 (15.9%)	716 (12.7%)	1067 (19.0%)	<.001
Non-Hispanic White	3864 (34.4%)	2079 (37.0%)	1785 (31.7%)	
Non-Hispanic Black	2295 (20.4%)	1569 (27.9%)	726 (12.9%)	
Other Hispanic	1189 (10.6%)	457 (8.13%)	732 (13.0%)	
Other Race	2115 (18.8%)	802 (14.3%)	1313 (23.4%)	
Marriage status				
Married/Living with partner	6226 (60.0%)	3101 (59.5%)	3125 (60.6%)	.261
Widowed/Divorced/Separated/Never married	4143 (40.0%)	2111 (40.5%)	2032 (39.4%)	
Alcohol				
No	9384 (91.0%)	4589 (89.3%)	4795 (92.6%)	<.001
Yes	930 (9.02%)	547 (10.7%)	383 (7.40%)	
Smoke				
Never	6886 (62.5%)	3187 (57.9%)	3699 (67.2%)	<.001
Former	1759 (16.0%)	998 (18.1%)	761 (13.8%)	
Current	2366 (21.5%)	1321 (24.0%)	1045 (19.0%)	
25(OH)D_2_ + D_3_ (nmol/L)	60.1 ± 24.9	59.2 ± 23.8	61.0 ± 26.0	<.001
25(OH)D_2_ (nmol/L)	3.14 ± 9.11	2.97 ± 8.90	3.31 ± 9.31	.043
25(OH)D_3_ (nmol/L)	57.0 ± 24.8	56.3 ± 23.6	57.7 ± 26.0	.001
Total calcium (mmol/L)	2.34 ± 0.09	2.35 ± 0.09	2.34 ± 0.09	<.001

Data were presented as mean ± SD or n (%).

BMI = body mass index.

### 3.2. Association of vitamin D and calcium with bone mineral content and density in the general population

As shown in Tables 3 and 4, multivariate linear regression analysis revealed a significant positive association between total vitamin D levels [25(OH)D_2_ + D_3_] and both BMD (β = 0.034, *P* = .001) and BMC (β = 87.87, *P* = .010), after adjusting for age, BMI, race, education background, marriage status, smoking, and alcohol consumption. Further analysis revealed differential effects of 25(OH)D_3_ and 25(OH)D_2_ in the general population. Specifically, 25(OH)D_3_ levels were negatively associated with both BMD and BMC (both *P* < .05), and a similar negative association was also observed for 25(OH)D_2_ (both *P* < .05). Additionally, total calcium levels were negatively associated with both BMD (β = −0.039, *P* < .001) and BMC (β = −109.28, *P* = .005), suggesting a potentially complex regulatory interaction between vitamin D and calcium in the general population. Furthermore, BMI was significantly positively correlated with both BMC and BMD (both *P* < .001), whereas age showed significant negative correlations with both parameters (both *P* < .001). Such outcomes suggested that individuals with higher BMI may have greater bone mineral levels, while aging may accelerate bone mineral reduction.

**Table 3:** Multivariate regression analysis of bone mineral density (BMD).

Variable	Estimate	Std. error	Statistic	*P*-value
25(OH)D_2_ + D_3_ (nmol/L)	0.0339	0.0102	3.305253	<.001
25(OH)D_2_ (nmol/L)	−0.0337	0.0103	−3.28405	.001
25(OH)D_3_ (nmol/L)	−0.0335	0.0102	−3.2684	.001
Total calcium (mmol/L)	−0.0392	0.0118	−3.33019	<.001
Age (yr)	−0.0013	0.0001	−14.518	<.001
BMI (kg/m^2^)	0.0027	0.0002	17.6151	<.001
Gender (Female)	−0.0711	0.0021	−34.2404	<.001
Education level				
Complete High school	−0.0015	0.0033	−0.4409	.659
>High school	0.01042	0.0029	3.5468	<.001
Race				
Non-Hispanic White	0.0156	0.0034	4.6274	<.001
Non-Hispanic Black	0.0857	0.0036	23.8450	<.001
Other Hispanic	0.0034	0.0040	0.8451	.398
Other Race	0.0024	0.0037	0.6442	.519
Marriage status				
(Widowed/divorced/separated/never married)	−0.0058	0.0021	−2.7222	.006
Alcohol (yes)	0.0072	0.0034	2.0957	.036
Smoke				
Former	−0.0033	0.0028	−1.1534	.249
Current	−0.0009	0.0026	−0.3488	.727

BMI = body mass index.

**Table 4:** Multivariate regression analysis of bone mineral content (BMC).

Variable	Estimate	Std. error	Statistic	*P*-value
25(OH)D_2_ + D_3_ (nmol/L)	87.8668	34.1196	2.5753	.01
25(OH)D_2_ (nmol/L)	−87.1233	34.1062	−2.5545	.01
25(OH)D_3_ (nmol/L)	−86.3103	34.0638	−2.5338	.01
Total calcium (mmol/L)	−109.2760	39.1166	−2.7936	.005
Age (yr)	−4.5840	0.3067	−14.9460	<.001
BMI (kg/m^2^)	18.1065	0.5099	35.5097	<.001
Gender (Female)	−522.4250	6.9069	−75.6382	<.001
Education level				
Complete high school	13.2241	10.9413	1.2086	.227
>High school	65.9971	9.7694	6.7555	<.001
Race				
Non-Hispanic White	134.7990	11.2287	12.0049	<.001
Non-Hispanic Black	339.6765	11.9595	28.4023	<.001
Other Hispanic	29.7802	13.4486	2.2144	.0268
Other Race	12.6491	12.3426	1.0248	.3055
Marriage status				
(Widowed/divorced/separated/never married)	−24.9796	7.0879	−3.5243	<.001
Alcohol (yes)	24.5630	11.4207	2.1508	.032
Smoke				
Former	5.5628	9.3972	0.5920	.554
Current	16.4636	8.7559	1.8803	.060

BMI = body mass index.

### 3.3. Analysis of the gender subgroup

To further clarify the regulatory role of gender in the associations of vitamin D and calcium intake with BMD and BMC, gender-stratified multivariate regression analyses were conducted. The analysis results revealed significant gender-specific differences in the effects of vitamin D and calcium on BMD and BMC.

Among males (Tables 5 and 6), total vitamin D [25(OH)D_2_ + D_3_] was significantly positively associated with BMD (β = 0.041, *P* = .011) and BMC (β = 126.21, *P* = .028). In contrast, 25(OH)D_2_ and 25(OH)D_3_ individually showed negative correlations with BMD and BMC (*P* < .05), suggesting a form-specific effect of vitamin D on BMD and BMC in males. Additionally, calcium intake was not significantly associated with either BMC or BMD among males (both *P* > .05), suggesting a potentially limited role of calcium in male bone marriage status. In contrast, BMI was positively linked to BMD and BMC (*P* < .001), while age was negatively associated (*P* < .001). Racial subgroup analysis disclosed that non-Hispanic Black males had significantly higher BMC and BMD levels compared with other races (*P* < .001).

**Table 5:** Multivariate regression analysis of bone mineral density (BMD) in male subgroups.

Variable	Estimate	Std. error	Statistic	*P*-value
25(OH)D_2_+ D_3_ (nmol/L)	0.0411	0.0162	2.5345	.011
25(OH)D_2_ (nmol/L)	−0.0409	0.0162	−2.5212	.012
25(OH)D_3_ (nmol/L)	−0.0404	0.0162	−2.4955	.013
Total calcium (mmol/L)	0.0215	0.0185	1.1578	.247
Age (yr)	−0.0009	0.0001	−6.5375	<.001
BMI (kg/m^2^)	0.0033	0.0003	12.9883	<.001
Education level				
Complete high school	−0.0035	0.0047	−0.7441	.457
>High school	0.0048	0.0042	1.1318	.258
Race				
Non-Hispanic White	0.0120	0.0050	2.4145	.016
Non-Hispanic Black	0.0945	0.0054	17.5791	<.001
Other Hispanic	0.0035	0.0061	0.5645	.572
Other Race	0.0041	0.0054	0.7454	.456
Marriage status				
(Widowed/divorced/separated/never married)	−0.0016	0.0032	−0.4838	.629
Alcohol (yes)	0.0127	0.0045	2.8586	.004
Smoke				
Former	−0.0063	0.0039	−1.6045	.1087
Current	−0.0031	0.0037	−0.8229	.411

BMI = body mass index.

**Table 6:** Multivariate regression analysis of bone mineral content (BMC) in male subgroups.

Variable	Estimate	Std. error	Statistic	*P*-value
25(OH)D_2_ + D_3_ (nmol/L)	126.2144	57.4021	2.1988	.028
25(OH)D_2_ (nmol/L)	−125.3600	57.3399	−2.1863	.029
25(OH)D_3_ (nmol/L)	−123.6000	57.3262	−2.1561	.031
Total calcium (mmol/L)	126.2144	57.4021	2.1988	.028
Age (yr)	−3.6439	0.5003	−7.2827	<.001
BMI (kg/m^2^)	22.4921	0.8938	25.1646	<.001
Education level				
Complete high school	2.8964	16.5400	0.1751	.861
>High school	48.4399	15.0268	3.2236	.001
Race				
Non-Hispanic White	132.4898	17.5833	7.5350	<.001
Non-Hispanic Black	396.1453	19.0146	20.8337	<.001
Other Hispanic	25.5553	21.6192	1.1821	.237
Other Race	23.6609	19.2246	1.2308	.219
Marriage status				
(Widowed/divorced/separated/never married)	−14.2559	11.4036	−1.2501	.211
Alcohol (yes)	29.8479	15.7449	1.8957	.058
Smoke				
Former	−12.9164	13.9392	−0.9266	.354
Current	12.9536	13.1729	0.9834	.326

BMI = body mass index.

Among females (Tables 7 and 8), total calcium levels were significantly negatively associated with BMC (β = −221.26, *P* < .001) and BMD (β = −0.073, *P* < .001), suggesting that bone health in females may be more sensitive to calcium homeostasis. However, unlike in males, total vitamin D and its subtypes were not significantly associated with either BMC or BMD in females (*P* > .05), suggesting that vitamin D may not be a critical determinant of BMC in females. Furthermore, BMI was significantly positively associated with both BMC and BMD in females (*P *< .001), whereas age exhibited a significant negative association (*P* < .001). Notably, marriage status was markedly associated with BMC in females (*P* < .01), while no such relationship was observed in males (*P* > .05), suggesting that psychosocial factors may play a role in bone health maintenance among females.

**Table 7:** Multivariate regression analysis of bone mineral density (BMD) in female subgroups.

Variable	Estimate	Std. error	Statistic	*P*-value
25(OH)D_2_ + D_3_ (nmol/L)	0.0219	0.0131	1.6695	.095
25(OH)D_2_ (nmol/L)	−0.0217	0.0131	−1.6538	.098
25(OH)D_3_ (nmol/L)	−0.0216	0.0131	−1.6542	.098
Total calcium (mmol/L)	−0.0726	0.0152	−4.7586	<.001
Age (yr)	−0.0016	0.0001	−12.9278	<.001
BMI (kg/m^2^)	0.0024	0.0002	12.2711	<.001
Education level				
Complete high school	0.0013	0.0046	0.2879	.773
>High school	0.0163	0.0041	4.0237	<.001
Race				
Non-Hispanic White	0.0185	0.0046	4.0507	<.001
Non-Hispanic Black	0.0781	0.0048	16.2713	<.001
Other Hispanic	0.0037	0.0053	0.7028	.482
Other Race	0.0020	0.0050	0.3921	.695
Marriage status				
(Widowed/divorced/separated/never married)	−0.0071	0.0028	−2.5117	.012
Alcohol (yes)	−0.0034	0.0055	−0.6206	.535
Smoke				
Former	−0.0007	0.0041	−0.1702	.865
Current	0.0010	0.0037	0.2524	.801

BMI = body mass index.

**Table 8:** Multivariate regression analysis of bone mineral content (BMC) in female subgroups.

Variable	Estimate	Std. error	Statistic	*P*-value
25(OH)D_2_ + D_3_ (nmol/L)	32.9744	40.0198	0.8240	.410
25(OH)D_2_ (nmol/L)	−32.2542	40.0169	−0.8060	.420
25(OH)D_3_ (nmol/L)	−32.2544	39.9454	−0.8075	.419
Total calcium (mmol/L)	−221.2560	46.5939	−4.7486	<.001
Age (yr)	−5.0667	0.3761	−13.4727	<.001
BMI (kg/m^2^)	15.4424	0.5859	26.3576	<.001
Education level				
Complete high school	23.3632	14.0889	1.6583	.097
>High school	82.2754	12.3863	6.6424	<.001
Race				
Non-Hispanic White	134.9855	13.9840	9.6529	<.001
Non-Hispanic Black	292.5143	14.6657	19.9455	<.001
Other Hispanic	33.1398	16.2784	2.0358	.042
Other Race	8.7444	15.4093	0.5675	.570
Marriage status				
(widowed/divorced/separated/never married)	−23.9083	8.6489	−2.7643	.006
Alcohol (yes)	15.6809	16.7567	0.9358	.349
Smoke				
Former	27.7444	12.4830	2.2226	.026
Current	23.0657	11.4376	2.0167	.044

BMI = body mass index.

Collectively, the above findings disclosed significant gender differences in the correlations of vitamin D and calcium intake with BMC and BMD. Additionally, age, BMI, race, and lifestyle factors may further modulate these associations, underscoring the importance of incorporating these interactions into future osteoporosis prevention strategies.

## 4. Discussion

This study systematically evaluated the correlations of total calcium and vitamin D levels with BMC and BMD, with a particular focus on gender-specific effects. Overall analysis revealed that both total calcium and vitamin D levels were significantly associated with bone health indicators (BMC and BMD), but distinct gender-specific patterns were observed. Briefly, vitamin D levels significantly influenced BMC and BMD in males, whereas total calcium had no significant effect; conversely, in females, total calcium was significantly associated with BMC and BMD, while vitamin D showed no clear association. These findings suggest the presence of significant gender-specific regulatory mechanisms in bone mineral metabolism and highlight the importance of incorporating gender as a critical factor in osteoporosis prevention and bone health interventions.

Calcium and vitamin D are essential nutrients for the accumulation and maintenance of bone minerals. Calcium constitutes the primary structural component of bone, whereas vitamin D contributes to bone mineral homeostasis indirectly by enhancing intestinal calcium absorption.^[21]^ Studies have stated that low calcium intake is associated with decreased BMD and increased risk of osteoporosis, while vitamin D deficiency may lead to secondary hyperparathyroidism, thereby accelerating bone loss.^[22]^ The results of this study displayed that both total calcium and vitamin D levels were significantly associated with BMC and BMD, which is largely consistent with previous research. However, further gender-stratified analyses revealed marked gender differences in this association, which may be attributable to gender-specific regulatory mechanisms in bone metabolism.

In the male population, total vitamin D levels demonstrated significant positive associations with BMC and BMD, while 25(OH)D_2_ and 25(OH)D_3_ individually were negatively correlated with both parameters. Such a complex pattern suggests that different forms of vitamin D may exert distinct biological effects. Evidence from prior research reveals that male bone health is more susceptible to regulation by both vitamin D and androgen (e.g., testosterone), with calcium intake exerting a comparatively weaker influence on bone density.^[16]^ Mechanistically, male individuals typically exhibit a higher bone turnover rate, and vitamin D levels may more directly influence the balance between bone formation and resorption. Androgen may impact bone metabolism by modulating the activity of the vitamin D receptor, thereby enhancing the protective effects of vitamin D on the bone. Additionally, calcium intake is generally sufficient in males, making the impact of supplemental calcium on bone density limited. Instead, the status of vitamin D is more likely to be a significant factor influencing bone health.^[23,24]^

By contrast, in the female population, the association between vitamin D levels and bone health indicators is not significant, while total calcium intake shows a strong positive correlation. Efficient intestinal calcium absorption is critically dependent on adequate active vitamin D. When vitamin D status is insufficient, calcium bioavailability declines, potentially diminishing the skeletal benefits of calcium supplementation.^[25]^ This relationship is particularly important in postmenopausal women, in whom estrogen deficiency accelerates bone turnover and heightens reliance on sufficient calcium availability.^[26]^ Therefore, calcium and vitamin D should be regarded as complementary rather than independent factors; optimizing calcium intake should be accompanied by screening for and correcting vitamin D insufficiency to achieve maximal skeletal protection. This may be closely related to the unique physiological changes in females, particularly the rapid bone loss associated with decreased estrogen levels after menopause.^[17,27]^ Estrogen is crucial in maintaining calcium homeostasis in bone metabolism. After a reduction in estrogen levels in females, the need for calcium significantly rises, making bone health in females more susceptible to the consequences of insufficient calcium intake.^[28,29]^ As such, for females, ensuring sufficient calcium intake may be more crucial than solely increasing vitamin D levels.

Furthermore, this study also revealed that BMI, age, and race have a significant impact on bone health. Individuals with higher BMI exhibit elevated bone mineral levels, likely due to the increased mechanical load resulting from weight gain, which stimulates bone formation. Other studies have stated that adipose tissue secretes estrogen-like hormones, which have a protective effect on bone density.^[30]^ Increasing age is significantly linked to a reduction in BMC and BMD, especially in the female population, further suggesting accelerated bone loss following menopause.^[31–33]^ Racial differences also significantly impact bone mineral levels, with non-Hispanic Black individuals having significantly higher BMC than other racial groups, which may be related to genetic background and lifestyle differences.

The results of this study provide valuable clinical insights for bone health management and nutritional intervention strategies, emphasizing the gender differences in the influence of nutritional factors on bone health. This suggests that future osteoporosis prevention strategies should account for gender-specific interventions. Moreover, the significant impact of factors such as age, race, and BMI on bone health indicates that personalized nutrition and health management strategies may yield more effective outcomes.

However, this study has certain limitations. First, the cross-sectional design restricts the ability to draw clear causal inferences, and future prospective cohort studies are needed for further validation. Second, this study did not separately analyze pre- and postmenopausal females, which may have masked the unique changes in bone metabolism in females after menopause. Finally, this study did not include key biomarkers of bone metabolism, such as parathyroid hormone and calcium-binding proteins. Future research could integrate additional biomarkers to further explore the specific mechanisms of bone mineral metabolism.

## 5. Conclusion

To sum up, this study has revealed significant gender differences in the effects of vitamin D and total calcium content on BMC and BMD. In males, vitamin D levels significantly influence BMC and BMD, while the effect of total calcium content is not prominent. In females, total calcium content has a significant impact on bone mineral levels, while vitamin D levels show no significant correlation. Furthermore, factors such as BMI, age, and race are closely linked to bone mineral status. These findings suggest that future bone health management and nutritional supplementation strategies should take into account the effects of gender, BMI, age, and race to develop more precise intervention measures.

## Author contributions

**Conceptualization:** Kai Chen, Pengli Zhang.

**Data curation:** Kai Chen, Pengli Zhang.

**Formal analysis:** Kai Chen, Pengli Zhang.

**Funding acquisition:** Kai Chen, Pengli Zhang.

**Investigation:** Kai Chen, Pengli Zhang.

**Methodology:** Kai Chen, Pengli Zhang.

**Project administration:** Kai Chen, Pengli Zhang.

**Resources:** Kai Chen, Pengli Zhang.

**Software:** Kai Chen, Pengli Zhang.

**Supervision:** Kai Chen, Pengli Zhang.

**Validation:** Kai Chen, Pengli Zhang.

**Visualization:** Kai Chen, Pengli Zhang.

**Writing – original draft:** Kai Chen, Pengli Zhang.

**Writing – review & editing:** Kai Chen, Pengli Zhang.
